# Supplementary material for: The interactome of intact mitochondria by cross-linking mass spectrometry provides evidence for coexisting respiratory supercomplexes
Source: Mol Cell Proteomics. 2017 Dec 8;17(2):216–32. doi: 10.1074/mcp.RA117.000470 (PMC5795388; doi:10.1074/mcp.RA117.000470)
Supplement: Supplemental Data [file supp_RA117.000470_133922_0_supp_23978_fzffwf.zip › spectra_annotation/mito_spectra_annotation/446-1-1-1-4-1.pdf]

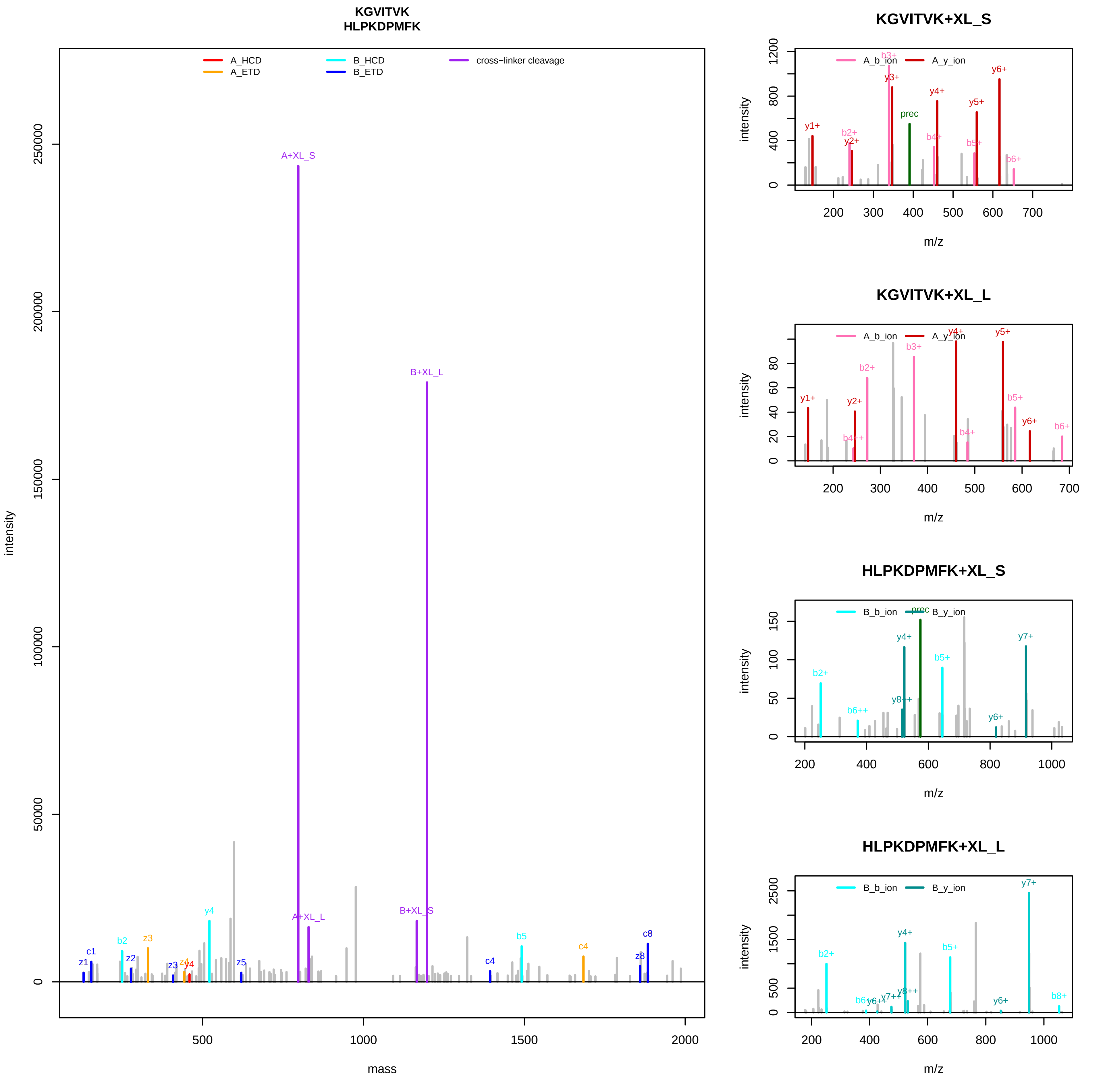
KGVITVK
HLPKDPMFK
A_HCD
A_ETD
B_HCD
B_ETD
cross−linker cleavage
A+XL_S
B+XL_L
A+XL_L
B+XL_S
c1 z1 b2 z2 z3 z3 z4 y4 y4 z5 c4 b5 c4 z8 c8
intensity
mass
0
50000
100000
150000
200000
250000
500
1000
1500
2000
KGVITVK+XL_S
A_b_ion
A_y_ion
y1+ b2+ y2+ b3+ y3+ prec y4+ b4+ y5+ b5+ y6+ b6+
m/z
KGVITVK+XL_L
y1+ b4++ y2+ b2+ b3+ y4+ b4+ y5+ b5+ y6+ b6+
HLPKDPMFK+XL_S
B_b_ion
B_y_ion
b2+ b6++ y8++ y4+ prec b5+ y6+ y7+
HLPKDPMFK+XL_L
b2+ b6++ y6++ y7++ y8++ y4+ b5+ y6+ y7+ b8+
